# Collagen cross-linking: when and how? A review of the state of the art of the technique and new perspectives

**DOI:** 10.1186/s40662-015-0030-6

**Published:** 2015-11-29

**Authors:** Leonardo Mastropasqua

**Affiliations:** Ophthalmology Department, Policlinico SS Annunziata, Center of Excellence and National High-Tech Center (CNAT) in Ophthalmology, University “G. d’Annunzio” of Chieti-Pescara, Via dei Vestini, 31 66100 Chieti, Italy

**Keywords:** Keratoconus, Ectasia, Collagen cross-linking, Transepithelial cross-linking, Collagen corneal cross-linking epithelium off, Collagen corneal cross-linking epithelium on, Transepithelial cross-linking with iontophoresis

## Abstract

Since the late 1990s corneal crosslinking (CXL) has been proposed as a new possibility to stop progression of keratoconus or secondary corneal ectasia, with the promising aim to prevent progressive visual loss due to the evolution of the pathology and to delay or avoid invasive surgical procedures such as corneal transplantation. The possibility of strengthening corneal tissue by means of a photochemical reaction of corneal collagen by the combined action of Riboflavin and ultraviolet A irradiation (UVA), radically modified the conservative management of progressive corneal ectasia. This is a review of the state of the art of CXL, reporting basic and clinical evidence. The paper describes basic principles, advantages and limits of different CXL techniques and possible future evolution of the procedure.

## Introduction

Corneal ectasia is a progressive corneal thinning associated with alterations of stromal collagen matrix resulting in irregular protrusion of the cornea. Primary forms include keratoconus, pellucid marginal degeneration and keratoglobus, while secondary forms are mainly related to refractive surgery [1].

Many studies on keratoconus epidemiology from different countries reported an incidence of 1.3 to 22.3 per 100 000 and a prevalence of 0.4 to 86 cases per 100 000 [2].

The incidence of corneal ectasia after refractive surgery is still unknown, but it has been estimated to be 0.04–0.6 % after laser in situ keratomileusis(LASIK) [3–5].

Post LASIK ectasia represents about 96 % of all secondary ectasias after refractive surgery, while 4 % are related to photorefractive keratectomy (PRK) surgery [6].

Keratoconus generally starts during the second decade of life with a variable rate of progression of corneal steepening that continues until the fourth decade, when the corneal shape generally becomes stable [7].

A conservative approach in the management of keratectasia initially involves spectacles and subsequently, contact lenses.

However, surgical intervention can be necessary when patients are contact lens intolerant or cannot achieve adequate vision correction. In patients with contact lens intolerance or poor visual recovery with contact lens use, implantation of intracorneal rings (ICRS) may improve the regularity of the corneal curvature, improving contact lens fitting and visual rehabilitation [8].

Alternatively, in advanced stages or in presence of central corneal scarring, corneal transplantation may represent the only viable treatment option [1]. In these cases the preferred procedure is deep anterior lamellar keratoplasty (DALK) or, alternatively, penetrating keratoplasty (PK).

Until recently, in the “pre-CXL era”, all of the treatment options for corneal ectatic diseases were aimed at only overcoming refractive limitations and not to impeding the underlying physiopathology [9].

Corneal crosslinking has grown from an interesting concept to its introduction in clinical practice in the late 1990s when it radically modified conservative management of progressive corneal ectasia with the possibility of strengthening corneal tissue [10].

## Review

### Basic principles of corneal crosslinking

The primary aim of corneal crosslinking is to stop the progression of corneal ectasia. To obtain a strengthening of corneal tissue, the use of riboflavin is combined with ultraviolet A irradiation (UVA). Riboflavin plays the role of a photosensitizer in the photopolymerization process and when combined with UVA irradiation, increases the formation of intrafibrillar and interfibrillar carbonyl-based collagen covalent bonds through a molecular process that has still not been completely elucidated [1].

It was shown that during the early aerobic phase of the process of crosslinking, riboflavin molecules are excited to a single or triplet state and stromal proteins undergo a photosensitized oxidation via interaction with reactive oxygen species [11]. During the second anaerobic phase, when oxygen is depleted, corneal stroma interacts with reactive species of radical ions. This photochemical reaction results in an increase of corneal rigidity, of collagen fiber thickness and of resistance to enzymatic degradation, with consequent decrease of stromal swelling and permeability maximally, above all in the anterior stroma [12].

### Basic research results

Currently, the photochemically induced effect of CXL in the cornea cannot be evidenced directly by staining methods or microscopic techniques. However, CXL induces several changes to collagen-containing tissue, from which indirect signs of the cross-linking effect can be deduced [9]. In fact, stress–strain measurements performed on human and porcine corneas documented an increased corneal rigidity after CXL treatment. The firming effect seems to be more evident in corneas with higher collagen content and in older tissue [13, 14]. Moreover, it has been reported that porcine crosslinked corneas showed a reduced tendency to swelling and hydration when compared to untreated controls [15]. Ex vivo studies on corneas of humans and rabbits indicated an increase of collagen fibers thickness after CXL treatment [16, 17]. Results of basic research studies showed that CXL procedure improve the corneal resistance to degradation processes mediated by pepsin, trypsin and collagenase with lengthening of the turnover time of the collagen [18].

### Indications for CXL

Not every cornea with keratoconus needs to undergo crosslinking. The main aim of CXL is to stop the progression of corneal ectasia, consequently the best candidates for this treatment are patients suffering from primary or post refractive surgery ectasia with documented progression of the disease. Although the criteria to classify ectasia as progressive have not been defined, changes in refraction, uncorrected visual acuity (UCVA), best-corrected visual acuity (BCVA), and topographical parameters are to be included. To date, published clinical studies indicated that CXL was used in cases of progression over a well-defined time period. In many reports, progression was defined as an increase in Kmax of 1 diopter (D) in 1 year, or a change in either myopia and/or astigmatism ≥3 D in 6 months, a mean central K-reading change ≥1.5 D observed in three consecutive topographies in 6 months, or a mean central corneal thickness decrease ≥5 % in three consecutive tomographies in the previous 6 months. Contraindications to undergoing standard CXL treatment are the presence of corneal thickness of less than 400 microns, prior herpetic infection, severe cornea scarring or opacification, history of poor epithelial wound healing, severe ocular surface disease, history of immune disorders, and pregnancy/breast-feeding [19–21].

### Standard procedure and clinical results

The standard Dresden protocol, as initially described by Wollensask et al. includes initial epithelial removal, the application of 0.1 % riboflavin solution for 30 min followed by 30 min of UVA irradiation with a wavelength of 370 nm and power of 3 mW/cm^2^ (5.4 J/cm^2^) [22].

A list of publications reporting the clinical results of traditional CXL procedures is shown in Table 1 [23–65]. In the last few years, several prospective and retrospective studies with a considerable follow-up period documented the effectiveness of the standard procedure in halting the progression of primary and secondary corneal ectasia, and in many cases, with an improvement of visual performance and topographical indexes.

**Table 1:** Outcomes reported in literature for standard epi-off CXL procedures (2010–2015)

Author (Year)	Follow-up	Results				Type of Study	Number of Eyes	Indication
		Kmax	K1/K2	Mean Keratometry	BCVA			
Lang S et al. (2015) [23]	3 years	D			-	RCS	29	Keratoconus
Poli M et al. (2015) [24]	6 years	S			I	PCS	36	Ectasia
Di Bernardo M et al. (2015) [25]	2 years	D			I	PCS	57	Keratoconus
McAnena L et al. (2015) [26]	3 years	S			I	RCS	25	Pediatric Keratoconus
Sedaghat M et al. (2015) [27]	1 year	S		D	I	PCS	97	Keratoconus
Khan WA (2015) [28]	3 years	D			I	PCS	71	Keratoconus
Yildirim A et al. (2014) [29]	42 months	D			I	RCS	20	Post LASIK ectasia
Kymionis GD et al. (2014) [30]	5 years		D		I	PCS	25	Keratoconus
Kumar Kodavoor S (2014) [31]	1 year	S			I	RCS	24	Pediatric Keratoconus
Viswanathan D et al. (2014) [32]	20 months		D		S	PCS	25	Pediatric Keratoconus
Goldich I et al. (2014) [33]	3 years	S			S	PCS	17	Keratoconus
Steinberg J et al. (2014) [34]	2 years	D			I	PCS	20	Keratoconus
Wittig-Silva C et al. (2014) [35]	3 years	D			I	RCT	100	Keratoconus
Elbaz U et al. (2014) [36]	1 year			S	S	PCS	9	Radial Keratotomy
Ghanem RC et al. (2014) [37]	2 years	D			I	PCS	42	Keratoconus
Toprak I et al. (2013) [38]	1 year	D			I	RCS	59	Keratoconus
Hashemi H et al. (2013) [39]	5 years	D			I	PCS	40	Keratoconus
Richoz O et al. (2013) [40]	2 years	D			I	RCS	26	Post LASIK/PRK ectasia
Ivarsen A et al. (2013) [41]	22 months	D			S	RCS	22	Keratoconus
Legare ME et al. (2013) [42]	2 years	S			S	RCS	39	Keratoconus
O’Brart (2013) [43]	4-6 years			S	S	RCS	30	Keratoconus
Arora R et al. (2012) [44]	12 months				I	PCS	15	Pediatric Keratoconus
Chatzis N et al. (2012) [45]	3 years	S			I	RCS	59	Pediatric Keratoconus
Vinciguerra R et al. (2013) [46]	4 years	D			I	RCS	400	Keratoconus
Viswanathan D et al. (2013) [47]	14 months	D			I	PIS	76	Keratoconus
Poli M. et al. (2013) [48]	3 years	S			S	PC	55	primary and secondary ectasia
Lamy R et al. (2013) [49]	2 years	D			I	PC	68	Keratoconus
Kranitz K et al. (2012) [50]	1 year		D		I	PC	40	Keratoconus
Guber I et al. (2013) [51]	1 year	S			I	PCS	33	Keratoconus
Vinciguerra P et al. (2012) [52]	2 years		K1 S/ K2 D		I	PCS	40	Pediatric Keratoconus
Caporossi A et al. (2012) [53]	3 years		D		I	PCS	152	Pediatric Keratoconus
Goldich I et al. (2012) [54]	2 years	D			I	PCS	14	Keratoconus
Asri D et al. (2011) [55]	1 year	S			S	PCS	142	Keratoconus
Fuentes-Paez G et al. (2012) [56]	6 months			S	I	PCS	7	Ectassia post RK
Kymionis GD et al. (2012) [57]	1 year			S	S	PCS	14	Keratoconus
Koller T et al. (2011) [58]	1 year	D			S	PCS	151	Keratoconus
Greenstain SA et al. (2011) [59]	1 year	D			S	RCT	71	Primary and secondary ectasia
Raiskup F et al. (2011) [60]	1 year	S			S	RCS	32	Keratoconus
Hersh P et al. (2011) [61]	1 year	S			I	RCT	71	Keratoconus
Salgado JP et al. (2011) [62]	1 year	S			S	PCS	22	Post LASIK ectasia
Caporossi A et al. (2010) [63]	4 years	D			I	PCS	44	Keratoconus
Vinciguerra P et al. (2009) [64]	2 years	D			I	PCS	28	Keratoconus
Vinciguerra P et al. (2010) [65]	1 year			D	I	PCS	13	Post LASIK/PRK ectasia

*S= * Stabilized, *D= * Significantly decreased, *I= * Significantly improved, *PCS=* Prospective case series, *RCS=* Retrospective case series, *RCT=* Randomized controlled trial, *PIS*= Prospective interventional study, *PC=* Prospective comparative

Most of the reports about clinical outcomes of standard epi-off CXL are prospective or retrospective case series. In the follow-up after treatment, the main parameters evaluated are the maximal keratometry (K_max_) and the best corrected visual acuity (BCVA). The follow-up periods ranged between one and six years. All authors reported stabilization or flattening of corneal keratometry and stabilization or improvement of visual acuity after standard epi-off procedure.

The small number of randomized controlled trials may affect the interpretation of these results. However, the results reported by Wittig-Silva et al. (2015) of 100 eyes with a three-year follow-up constitute an important milestone that confirms the effectiveness of epi-off technique in stabilizing keratoconus progression [35].

### Limits and complications of standard procedure

#### Treatment failure

Treatment failure that occurs in 8.1–33.3 % of the cases is usually defined as continued progression with an increase in maximum K readings of 1.0 D over the preoperative value [66].

Poli et al. recently reported a failure rate of 11 % during a follow-up period of six years. Keratoconus worsening was considered if patients presented an increase of more than 0.1 in logMAR uncorrected and best corrected visual acuity and/or an increase of keratometric values by more than 0.75 D during the follow-up [24].

After standard CXL procedure, corneal haze is a relatively common complication reported by 10-90 % of patients. However, to date the etiology and the natural course of clinical corneal haze after epi-off procedure has not been clearly defined [67, 68]. In vivo confocal microscopy showed an increased stromal reflectivity associated to edema and keratocyte activation mainly evident 3–6 months after treatment, while in the late postoperative period, anterior and intermediate stromal layers showed a reduction of cellular density and fibrosis of extracellular matrix [69].

Several cases of infective keratitis following CXL treatment have been described including bacterial, protozoal, herpetic, and fungal keratitis [70].

The rare serious adverse events following traditional CXL that have been reported included diffuse lamellar keratitis at LASIK interface, corneal melting and persistent corneal edema due to endothelial failure [71–73].

### Introduction of epi-on technique

The diffusion process of riboflavin in the stroma is limited by corneal epithelial tight junctions [74, 75], but epithelial debridement is considered the cause of the most important complications after CXL treatment such as intraoperative and postoperative pain, infective keratitis and abnormal wound-healing response [76, 77]. Riboflavin penetration through the epithelium can be increased by different strategies such as changing the physicochemical properties of the riboflavin molecule by adding chemical enhancers in the riboflavin formulation [78] or performing a mechanical disruption of corneal epithelium [79]. An in vivo confocal microscopy study reported that by increasing the duration of riboflavin application up to two hours, the obtained depth of CXL effect is similar to that achieved with standard epi-off technique [80].

Although the complication rate in patients treated with transepithelial CXL was reported to be low [69], so was the effectiveness of this technique (Table 2) [81–89]. Thus, its utility is still a matter of debate.

**Table 2:** Outcomes reported in literature for epi-on CXL procedures (2010–2015)

Author (Year)	Follow-up	Results			Design	Number of Eyes	Indication
		Kmax	Mean Keratometry	BCVA			
Lensiak SP et al. (2014) [81]	6 months	S		S	PCS	25	Keratoconous
De Bernardo M et al. (2014) [82]	6 months	S		I	PCS	36	Keratoconus
Khairy HA et al. (2014) [83]	1 year	D		S	PCS	32	Keratoconus
Salman AG (2013) [84]	1 year	S		S	PC	22	Pediatric Keratoconus
Caporossi A et al. (2013) [85]	2 years	W		S	PCS	26	Keratoconus
Buzzonetti L et al. (2012) [86]	18 months		W	I	PCS	13	Pediatric Keratoconus
Spadea L et al. (2012) [87]	1 year	D		I	PCS	16	Keratoconus
Filippello M et al. (2012) [88]	18 months	D		I	PC	20	Keratoconus
Leccisotti A et al. (2011) [89]	1 year	S		I	RCT	51	Keratoconus

*S*= Stabilized, *D= * Significantly decreased, *I= * Significantly improved, *W= * worsened, *PCS=* Prospective case series, *RCT=* Randomized controlled trial, *PC=* Prospective comparative

### Iontophoresis

A novel approach to enhance riboflavin penetration is based on iontophoresis, a non-invasive system aimed to enhance the delivery of charged molecules into tissues using a small electric current [90]. Riboflavin, in the formulation used for iontophoresis, is negatively charged. It has been shown that an iontophoresis imbibition lasting five minutes achieves a sufficient riboflavin concentration in the corneal stroma for CXL treatment, with the advantage of shortening the imbibition time while preserving epithelial integrity [9].

Numerous ex vivo studies confirmed the effectiveness of iontophoresis imbibition in obtaining an adequate riboflavin concentration into the stroma and the induction of important biomolecular and structural modifications of corneal tissue [90–92]. Ex vivo biomechanical studies on rabbit and human cadaveric corneas showed that transepithelial crosslinking with iontophoresis imbibition induced an increase of the biomechanical resistance of human cornea comparable to that obtained with the standard crosslinking procedure [93, 94].

Preliminary clinical results of iontophoresis assisted corneal CXL are promising. The technique halts keratoconus progression without significant complications (Table 3) [95–97] however, longer follow-up and studies with larger patient populations are needed to assess the real effectiveness of this technique.

**Table 3:** Outcomes reported in literature for iontophoresis assisted corneal CXL procedures (2014–2015)

Author (Year)	Follow-up	Results		Type	Number of Eyes	Indication
		Kmax	BCVA			
Buzzonetti L et al. (2015) [95]	15 months	S	I	PCS	14	Pediatric Keratoconus
Bikbova G et al. (2014) [96]	1 year	D	I	PCS	22	Keratoconus
Vinciguerra P et al. (2014) [97]	1 year	S	I	PCS	20	Keratoconus

*S= * Stabilized, *D*= Significantly decreased, *I= * Significantly improved, *PCS=* Prospective case series

### Accelerated corneal crosslinking

Accelerated CXL was introduced in clinical practice in order to shorten the time required for a CXL procedure. This technique is based on the Bunsen-Roscoe law of photochemical reciprocity. That is, the same photochemical effect can be achieved with reducing the irradiation interval provided that the total energy level is kept constant by a corresponding increase in irradiation intensity [1]. Currently, commercially available ultrafast devices can achieve an irradiance intensity of 43 mW/cm^2^. Using this setting, a total treatment time of two minutes is required to achieve a standard Dresden protocol energy dose of 3.4 J or a radiant exposure of 5.4 J/cm^2^ [1]. Several recent in vivo studies using different protocols showed the procedure to be safe and effective in stopping ectasia progression (Table 4) [98–105].

**Table 4:** Outcomes reported in literature for accelerated corneal CXL procedures (2014–2015)

Author (Year)	Tecnique	Follow-up	Results			Type	Number of Eyes	Indication
			Kmax	K1/K2	BCVA			
Chan TC et al. (2015) [98]	Accelerated	1 year	S		S	PCS	25	Keratoconus
Ozgurhan EB et al. (2015) [99]	Accelerated	1 year	R		I	PCS	34	Keratoconus
Marino GK et al. (2015) [100]	Accelerated	2 years	S		S	PCS	40	Post LASIK ectasia
Waszczykowska A (2015) [101]	Accelerated	2 years	S		S	PCS	16	Keratoconus
Ozgurhan EB et al. (2014) [102]	Accelerated	2 years		D	I	RCS	44	Pediatric Keratoconus
Shetty R et al. (2014) [103]	Accelerated	2 years	-		I	PCS	18	Keratoconus
Elbaz U et al. (2014) [104]	Accelerated	1 year	S		S	PCS	16	Keratoconus
Cinar Y et al. (2014) [105]	Accelerated	6 months	D		I	PCS	23	Keratoconus

*S= * Stabilized, *D= * Significantly decreased, *I*= Significantly improved, *PCS=* Prospective case series, *RCS=* Retrospective case series

Comparative studies of the effectiveness of the different CXL procedures are described in Table 5 [106–115]. Surgical protocols reported are very different and unlikely comparable. Also, the follow-up periods are very limited. Therefore, it is very difficult to deduce reliable conclusions. It seems likely that transepithelial CXL, although associated with a lower complication rate, has a lower therapeutic effect than standard CXL and would be ideal for patients with thin corneas, uncooperative individuals, or those with uncertain documented progression. Iontophoresis assisted CXL is a promised technique that could obtain clinical effects similar to those obtainable with a standard technique while maintaining the advantages of epithelium preservation. However, while the basic research results are evident, clinical outcomes are still poor. Accelerated CXL seems to represent a valid strategy to shorten the long treatment time, however the extreme variability of the protocols proposed has not been supported by adequate safety assessment. Well-designed randomized controlled trials comparing traditional CXL and all the alternative procedures are required in order to establish which is the ideal protocol for obtaining the best clinical outcomes and complication profile.

**Table 5:** Outcomes reported in literature comparing different CXL procedures (2013–2015)

Author (Year)	Techniques compared	Follow-up	Results		Type	Number of Eyes	Indication
			Kmax	BCVA			
NG AL et al. (2015) [106]	Standard vs accelerated	1 year	Reduction Kmax in standard group	Improvement BCVA in standard CXL	CIS	26	Keratoconus
Shetty R et al. (2015) [107]	Standard vs Different Accelerated Protocols	1 year	Improvement Kmax in standard and accelerated group	Improvement BCVA in Standard and accelerated CXL	PR	138	Keratoconus
Rossi S et al. (2015) [108]	Standard vs TE	1 year	Improvement Kmax in standard and TE CXL	Improvement BCVA in standard and TE CXL	PC	20	Keratoconus
Brittingham et al. (2014) [109]	Standard vs accelerated	1 year	Stabilization K max in standard CXL		RCS	131	Keratoconus
Hashemian H et al. (2014) [110]	Standard vs Accelerated	15 months	Improvement Kmax in standard and accelerated CXL	Improvement BCVA in Standard and accelerated CXL	RCT	153	Keratoconus
Sherif AM (2014) [111]	Standard vs Accelerated	1 year	Stabilization Kmax	Improvement BCVA in Standard and accelerated CXL	RCT	25	Keratoconus
Stojanovic A et al. (2014) [112]	Standard vs TE	1 year	Stabilization Kmax for standard and TE CXL	Improvement BCVA in standard and TE CXL	RCT	40	Keratoconus
Tomita M et al. (2014) [113]	Standard vs Accelerated	1 year	Improvement Kmax in standard and accelerated CXL	Improvement BCVA in Standard and accelerated CXL	PC	48	Keratoconus
Cinar Y et al. (2014) [114]	Standard vs Accelerated	6 months	Improvement Kmax in standard and accelerated CXL	Improvement BCVA in Standard and accelerated CXL	PC	26	Keratoconus
Magli A et al. (2013) [115]	Standard vs TE	1 year	Improvement K max in standard and TE CXL	Improvement BCVA in standard and TE CXL	PC	37	Keratoconus

*CIS=* Comparative Interventional Study, *PR=* Prospective randomized, *RCS*= Retrospective case series, *RCT=* Randomized controlled trial, *PC*= Prospective comparative

### Combined treatments

#### CXL and photorefractive keratectomy

Keratoconus has always been considered a contraindication for PRK. However, during the last few years, the idea of performing PRK in patients with stable keratoconus has been proposed. Consequently, the possibility of combining CXL and PRK was introduced in clinical practice [116–120]. Several clinical reports demonstrated stability in corneas that had undergone a combination of CXL and PRK, either sequentially or combined. Patients experienced improvement in spherical equivalent (SE), defocus equivalent, uncorrected and best corrected visual acuity, high order aberrations and Kmax with stabilization of keratoconus progression during a follow up period of 12–24 months [121–124].

The timing of the ablation treatment and CXL as well as the interval between the two procedures has become topics of discussion. It was reported that patients who underwent both PRK and CXL procedures in the same day obtained better clinical and topographical results with a lower rate of corneal haze, compared to patients treated sequentially [125]. This may be related to the unpredictable refractive outcomes when excimer ablation is performed on cross-linked tissue [126]. However, performing both procedures concurrently in the same day may cause an irregular healing process with the formation of persistent stromal haze, probably related to keratocyte activation, which permanently affects visual performance [127, 128].

#### CXL and intracorneal rings

Studies reported that CXL halts keratoconus progression, but the overall results in terms of visual rehabilitation were insufficient. On the other hand, intracorneal ring segments (ICRS) produced rapid and substantial improvements of visual parameters but do not stop progression. Theoretically, a combination of these two procedures can produce better results [1].

Several studies reported concordant results confirming that combining CXL and ICRS implantation improved uncorrected and best corrected visual acuity, refraction, and keratometry during variable follow-up periods (7–12 months) [129–131]. One study reported no difference in topographical or visual outcome after ICRS or ICRS combined with CXL. Thus, the real effect of the combined treatment to the keratoconus progression is still unclear [132].

Moreover, it was reported that after one or both ring explantations, the refractive effects may be stable or reversible while the topographic changes seems to be maintained [133]. Therefore, while collagen crosslinking can be performed before, in conjunction with, or after ICRS implantation, the ideal method for combining these two treatments is still undefined [1].

### Alternative uses of corneal crosslinking

#### Infections

Crosslinking has an antimicrobial effect inherent to UV light interacting with riboflavin as the chromophore. In fact, UV irradiation is used as an antimicrobial procedure for disinfecting water, surfaces and air. It damages both the DNA and RNA of pathogens including bacteria and viruses, and renders them inactive [134].

Additionally, the photoactivated riboflavin seems to produce an antimicrobial effect. In fact, the use of riboflavin as a photosensitizer to inactivate pathogens in plasma, platelet, and red cell products has been described [135].

Due to its nucleic acid specificity and its limited tendency toward indiscriminate oxidation, riboflavin was hypothesized as a photosensitizer for the inactivation of pathogens in infective keratitis. It was reported that riboflavin activated by UVA showed an antimicrobial effect on agar plates inoculated with *Pseudomonas aeruginosa*, *Staphylococcus aureus*, *Staphylococcus epidermidis*, *Streptococcus pneumoniae,* and *Candida albicans*. The inhibition of microbial growth was significantly higher in plates treated with UVA activated riboflavin than in those treated with UVA light alone. However, riboflavin alone did not show any significant bactericidal effect [136].

The first reported use of CXL in infective keratitis was in 2008, when Iseli at al. reported healing 4 out of 5 cases of mycobacterial and fungal corneal melting unresponsive to conventional therapy, treated with the standard Dresden protocol [137]. In 2013, Alio et al. in a systematic review and meta-analysis reported similar results [138]. In 2014, Said et al. reported a large prospective clinical trial on infective keratitis comparing 21 eyes treated with CXL in addition to antimicrobial therapy in 19 eyes that received only antimicrobial therapy. They did not find a significant difference between both groups in terms of healing time and final visual acuity. Three patients treated with antimicrobial therapy alone experienced corneal perforation and one an infection relapse while no significant complications occurred in CXL group. The authors conclude that CXL could serve as a valuable adjuvant therapy and may reduce or avoid severe complications preventing the need for emergency keratoplasty [139].

#### Pseudophakic bullous keratopathy

In case of corneal edema due to endothelial failure, it has been shown that CXL effect increases corneal resistance to swelling processes. In fact CXL increases the interfiber collagen connections and it’s difficult for stromal fluid to separate collagen lamellae and create a potential space for edema accumulation . Therefore, the use of corneal CXL was proposed as an alternative approach for the management of pseudophakic bullous keratopathy (PBK) with the aim to reduce ocular discomfort, improve visual acuity, and delay the need for keratoplasty [140].

Clinical studies evaluating the effectiveness of corneal CXL in the treatment of PBK reported a significant improvement in corneal transparency, corneal thickness, and ocular pain one month postoperatively. However, CXL did not seem to have a long-lasting effect over six months in decreasing pain and maintaining corneal transparency [141, 142].

## Conclusions

At the light of this review we can conclude that there is still much to understand about the real modification of corneal collagen structure after the photochemical CXL reaction. Moreover the constant aim of basic and clinical research today is to identify the best strategies and combination of imbibition and irradiation that can lead to the better clinical efficacy together with the maximum safety of the treatment.

## Abbreviations

CXL: Corneal crosslinking
DALK: Deep anterior lamellar keratoplasty
UVA: Ultraviolet A irradiation
UCVA: Uncorrected visual acuity
BCVA: Best-corrected visual acuity
D: Diopter
PRK: Photorefractive keratectomy
SE: Spherical equivalent
ICRS: Intracorneal ring segments
PBK: Pseudophakic bullous keratopathy
